# Reducing mitochondrial bound hexokinase II mediates transition from non-injurious into injurious ischemia/reperfusion of the intact heart

**DOI:** 10.1007/s13105-017-0555-3

**Published:** 2017-03-03

**Authors:** Rianne Nederlof, Ebru Gürel-Gurevin, Otto Eerbeek, Chaoqin Xie, G. Sjoerd Deijs, Moritz Konkel, Jun Hu, Nina C. Weber, Cees A. Schumacher, Antonius Baartscheer, Egbert G. Mik, Markus W. Hollmann, Fadi G. Akar, Coert J. Zuurbier

**Affiliations:** 1 Laboratory of Experimental Intensive Care and Anesthesiology (L.E.I.C.A.), Department of Anesthesiology, Academic Medical Center, Meibergdreef 9, 1105 AZ Amsterdam, The Netherlands; 2 Department of Biology, Faculty of Science, University of Istanbul, 34134, Fatih, Istanbul, Turkey; 3 Department of Physiology, Academic Medical Center, Meibergdreef 9, 1105 AZ Amsterdam, The Netherlands; 4 Cardiovascular Research Center, Mt. Sinai School of Medicine, New York, NY 10029 USA; 5 Department of Clinical and Experimental Cardiology, Academic Medical Center, Meibergdreef 9, 1105 AZ Amsterdam, The Netherlands; 6 Department of Anesthesiology, Laboratory of Experimental Anesthesiology, Erasmus University Medical Center Rotterdam, ’s Gravendijkwal 230, 3015 CE Rotterdam, The Netherlands

**Keywords:** Ischemia/reperfusion injury, Hexokinase, Reactive oxygen species, Oxygen consumption

## Abstract

Ischemia/reperfusion (I/R) of the heart becomes injurious when duration of the ischemic insult exceeds a certain threshold (approximately ≥20 min). Mitochondrial bound hexokinase II (mtHKII) protects against I/R injury, with the amount of mtHKII correlating with injury. Here, we examine whether mtHKII can induce the transition from non-injurious to injurious I/R, by detaching HKII from mitochondria during a non-injurious I/R interval. Additionally, we examine possible underlying mechanisms (increased reactive oxygen species (ROS), increased oxygen consumption (MVO_2_) and decreased cardiac energetics) associated with this transition. Langendorff perfused rat hearts were treated for 20 min with saline, TAT-only or 200 nM TAT-HKII, a peptide that translocates HKII from mitochondria. Then, hearts were exposed to non-injurious 15-min ischemia, followed by 30-min reperfusion. I/R injury was determined by necrosis (LDH release) and cardiac mechanical recovery. ROS were measured by DHE fluorescence. Changes in cardiac respiratory activity (cardiac MVO_2_ and efficiency and mitochondrial oxygen tension (mitoPO_2_) using protoporphyrin IX) and cardiac energetics (ATP, PCr, ∆G_ATP_) were determined following peptide treatment. When exposed to 15-min ischemia, control hearts had no necrosis and 85% recovery of function. Conversely, TAT-HKII treatment resulted in significant LDH release and reduced cardiac recovery (25%), indicating injurious I/R. This was associated with increased ROS during ischemia and reperfusion. TAT-HKII treatment reduced MVO_2_ and improved energetics (increased PCr) before ischemia, without affecting MVO_2_/RPP ratio or mitoPO_2_. In conclusion, a reduction in mtHKII turns non-injurious I/R into injurious I/R. Loss of mtHKII was associated with increased ROS during ischemia and reperfusion, but not with increased MVO_2_ or decreased cardiac energetics before damage occurs.

## Introduction

The best method to reduce cardiac injury after ischemic heart disease, like a myocardial infarction, is to shorten the ischemic period by quickly restoring reperfusion. However, reperfusion itself can paradoxically cause further injury (ischemia/reperfusion (I/R) injury) and increase infarct size. This can lead to heart failure, which is currently the highest contributor to death caused by cardiovascular disease. If, however, the size of the infarct is decreased, the degree of heart failure will also decrease. Therefore, unravelling the important determinants that dictate when injury starts to develop during I/R is crucial in devising new I/R therapies.

Previous research has shown that reduced binding of the glycolytic enzyme hexokinase II (HKII) to the mitochondria (mtHKII) exacerbates the injury of an already injurious I/R insult [28, 33, 39]. It is, however, unknown whether reducing mtHKII during a normally non-injurious I/R episode can transform it into an injurious episode. In that case, the amount of mtHKII is an important determinant of turning reversible, non-injurious I/R into one that is injurious. The underlying mechanisms associated with this transition can then allude to the important processes causing this injury. Proposed physiological mechanisms linking decreased mtHKII to injury are increased reactive oxygen species (ROS) production, increased mitochondrial respiration and decreased high-energy phosphates (phosphocreatine (PCr)) and ATP [7, 22, 25, 28].

Decreasing mtHKII was reported to increase ROS production in isolated cardiac and brain mitochondria [7, 32], in neonatal cardiomyocytes [39] and following an injurious I/R period in the isolated rat heart [25]. Whether loss of mtHKII can already induce ROS production during a much shorter non-injurious period of ischemia in the isolated rat hearts is, however, unknown. It has now become clear that increased activity of the mitochondrial respiratory chain contributes to I/R injury [3, 30]. We have shown that non-injurious ischemia, like ischemic preconditioning (IPC), increases mtHKII [12, 41] and slows down mitochondrial activation [43], but only with glucose present [42], suggesting a necessity for HK. In addition, structurally obstructing HKII attachment to mitochondria obliterated IPC cardioprotection [1]. These findings are commensurate with the glucose dependency of IPC [21]. From these different observations, we hypothesise that decreasing mtHKII may result in increased mitochondrial activity, i.e. oxygen consumption, which then can contribute to increased I/R injury.

Finally, hexokinase binds to the voltage-dependent anion channel (VDAC). VDAC is responsible for the transport of adenine nucleotides and other metabolites across the outer mitochondrial membrane (OMM) [6]. Its close proximity to ATP/ADP exchange can give HKII an important role in cardiac energetics (ATP and PCr). However, it is unknown whether loss of mtHKII in the intact heart before the ischemic period affects cardiac energetics. It is possible that decreasing mtHKII results in impaired cardiac energetics before ischemia, in which impairment may then contribute to the development of injurious I/R. Additionally, when reducing mtHKII does indeed result in injurious I/R, cardiac energetics after ischemia is then anticipated to be reduced as a consequence of the increased injury (but the decreased energetics can then not be viewed as a driver of augmented I/R injury, because the changes occur after the I/R insult).

Therefore, in this study, we tested the hypothesis that decreasing mtHKII mediates the transition from non-injurious towards injurious cardiac I/R and that this transition is associated with increased ROS production, decreased PCr and increased oxygen consumption.

## Methods

### Animals

Animal experiments were approved by the animal Ethical Committee of the University of Amsterdam. Male Wistar rats (324 ± 3 g) were obtained from Charles River. Animals were housed 2–4 per cage, fed ad libitum and were subjected to a 12-h dark/12-h light cycle.

### Heart perfusion

**Fig. 1:**
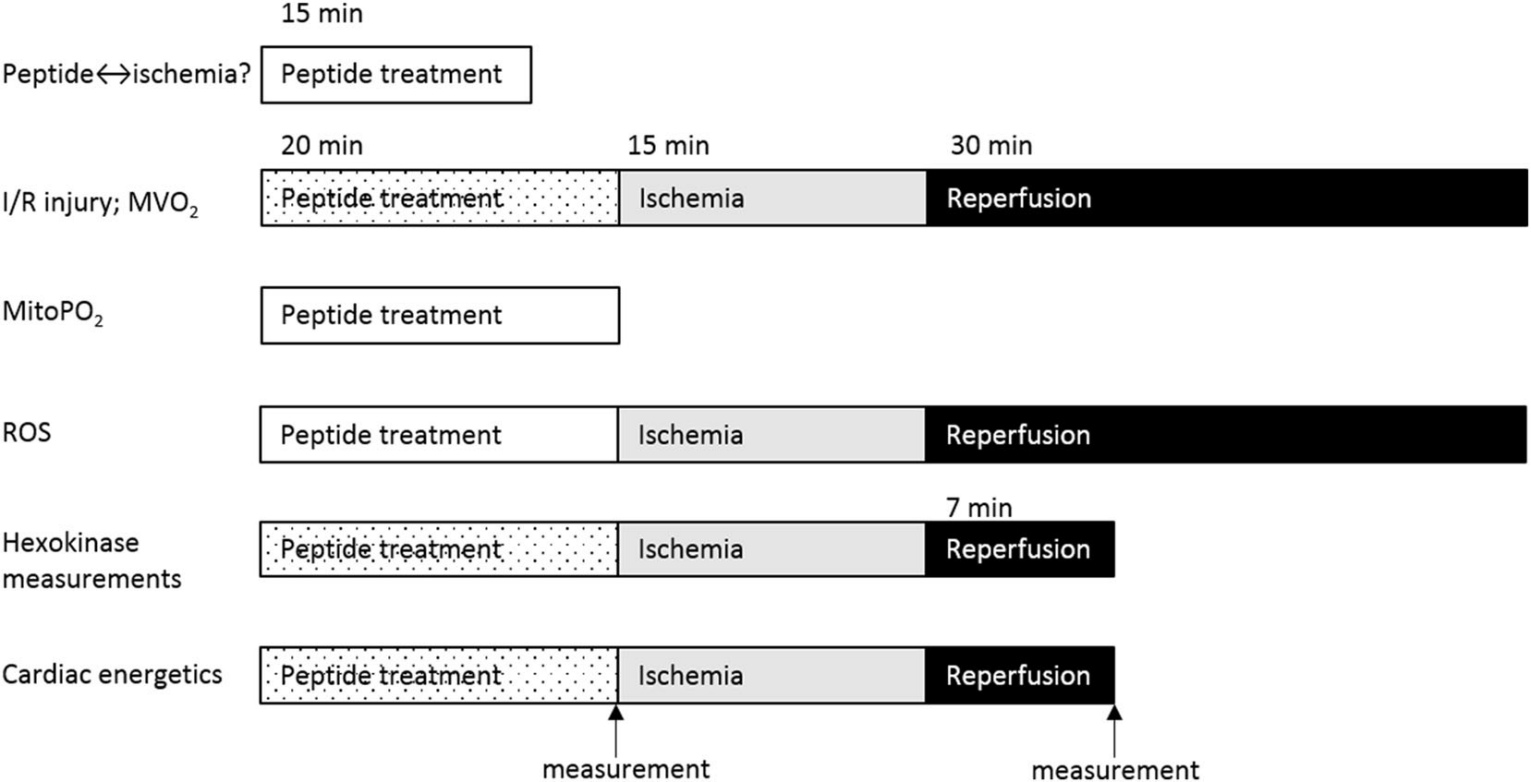
Perfusion protocols used for peptide effects on ischemia development, I/R injury and cardiac oxygen consumption, mitochondrial PO_2_ within the intact heart, ROS production, mitochondria-hexokinase binding and cardiac energetic parameters (PCr, ATP). Hearts were perfused with saline, TAT-only or TAT-HK peptide for 15–20 min after which some hearts were exposed to 15-min ischemia and 7- or 30-min reperfusion. I/R, cardiac energetics and hearts for hexokinase measurements were paced at 320 bpm during peptide treatment (*dotted*). NADH experiments were performed during 15-min peptide treatment. Cardiac energetics was measured at the end of peptide treatment and at 7-min reperfusion

The different perfusion protocols are presented in Fig. 1. Rats were anaesthetised with pentobarbital (60 mg/kg), tracheotomy was performed and mechanical ventilation started. Intravenous heparin (150 IU) was administered after opening of the thorax. The aorta was cannulated in situ, perfusion started and the heart was excised. Hearts were Langendorff perfused at a constant flow (initial perfusion pressure 80 mmHg) at 37 °C with Krebs-Henselheit buffer (KHB) containing the following (mM): 118 NaCl, 0.5 EDTA, 4.7 KCl, 2.25 CaCl_2_, 1.2 MgSO_4_, 25 NaHCO_3_, 1.2 KH_2_PO_4_ and 11 glucose gassed with 95% O_2_/5% CO_2_. End-diastolic pressure was set at ∼3–5 mmHg using a water-filled polyethylene balloon inserted into the left ventricular cavity. Hearts were continuously submerged in 37 °C KHB.

#### Peptide treatment

After a stabilisation period, hearts were treated with saline (control group 1), 1 μM TAT-only (control group 2), 200 nM TAT-HKII or 1 μM TAT-HKII. TAT-HKII (MIASHMIACLFTELN(β-Ala)GYGRKKRRQRRRG-amide) and TAT-only (GYGRKKRRQRRRG-amide) are soluble peptides produced by Pepscan Presto (Lelystad, The Netherlands). TAT-HKII translocates HKII from the mitochondria to the cytosol [5, 25, 33]. Peptides were dissolved in KHB solution and administered through a side-arm connected to a mixing chamber above the heart at 1% of total perfusion flow.

### Lactate production and NADH epifluorescence

During the execution of the present study, new research suggested that high doses of TAT-HKII may induce ischemia in the intact heart [26]. Therefore, NADH fluorescence of the surface of the heart and effluent lactate were evaluated as direct indices of ischemia in the 1 μM TAT-HKII group as described before [23] and compared to the control (2.5 μM TAT-HKII and 200 nM TAT-only) values of that study.

### Ischemia/reperfusion experiments

Rat hearts were perfused as described above. Hearts were paced at 320 bpm, and oxygen consumption (MVO_2_) was measured using an oxygen sensor (World Precision Instruments) in the left pulmonary artery. After the 20-min stabilisation period, hearts were exposed to 20-min peptide treatment, followed by 15-min global no-flow ischemia and 30-min reperfusion. During ischemia, hearts were submerged in KHB gassed with 95% N_2_/5% CO_2_, and pacing was stopped. During reperfusion, flow was slowly increased to initial flow within the first minute of reperfusion. Venous effluent was sampled at 5-, 10-, 20- and 30-min reperfusion for determination of lactate dehydrogenase (LDH) leakage as index of cell death.

### Mitochondrial oxygen tension

Mitochondrial oxygen tension (mitoPO_2_) was measured in a separate series of Langendorff perfused hearts using protoporphyrin IX (PpIX) as described before [18, 19]. In short, rats were injected with ALA (5-aminolevulinic acid hydrochloride) 2 h before the heart was dissected to enhance PpIX. Langendorff perfusion was performed as described above, but without pacing of the heart. Hearts were exposed for 20 min to saline or 200 nM TAT-HKII (*n* = 5 per group). PpIX was excited by a laser, and its delayed fluorescence was measured at 0-, 5-, 10-, 15- and 20-min peptide infusion.

### ROS measurements

In a dedicated series of experiments for ROS measurements, Langendorff perfused rat hearts (*n* = 6 per group) were exposed to 20-min treatment with 1 μM TAT-only or 200 nM TAT-HKII after which they were subjected to 15-min ischemia followed by 30-min reperfusion. An additional small series (*n* = 3) examined saline effects on ROS. Relative changes in O_2_ levels were measured using DHE fluorescence as described before [2].

### Determination of mitochondrial hexokinase

Rat hearts (*n* = 6 per group) were Langendorff perfused and exposed to saline, 1 μM TAT-only or 200 nM for 20 min. Then, hearts were exposed to 15-min no-flow ischemia. After 7-min reperfusion, hearts were homogenised as described before [41]. The 7-min time-point was chosen, because at that time-point, the peak in ROS levels was observed. Hearts were minced and homogenized on ice in ice-cold homogenisation medium (0.25 M sucrose, 0.02 M HEPES and 1 mM dithiothreitol, pH 7.4). Part of the homogenate was immediately centrifuged at 10,000*g* for 10 min at 4 °C. The pellet, representing the mitochondrial enriched fraction, was quickly frozen and stored at −80 °C until western blotting and determination of HK activity. We have previously shown that just one 10,000*g* centrifugation step results in a 2.5 times mitochondrial enrichment of the pellet as compared to whole homogenate [12]. Then, the mitochondrial enriched fraction was dissolved in 1-mL homogenisation medium with 50-μL 10% Triton X-100 and 10-μL 100 mM glucose-6-phosphate (to promote release of HK), stirred and incubated 15 min at room temperature and sonificated two times for 5 s. After centrifugation for 1 min at 10,000*g*, the amount of protein was determined in the supernatant using the Bradford protein assay.

#### Western blot

The amount of mtHKII was determined using western blot. For western blot, equal amounts of protein were loaded on a 4–12% gradient gel (Biorad), electrophoresed and transferred to a polyvinylidene membrane [24]. Membranes were incubated overnight with the primary antibody against HKII (1:1000; Cell Signalling C64G5) and the mitochondrial marker COX IV (1:9000; Cell Signalling 4844). Immunoreactive bands were visualized by the Odyssey system and quantified using the Odyssey IR Manager. All samples were analysed on the same blot.

### Lactate dehydrogenase and hexokinase activity

LDH activity in the effluent was determined as a measure of cell death, since other studies have shown a good correlation between LDH activity and TTC staining [20, 29]. It was determined spectrophotometrically by measuring NADH oxidation at 340 nm after addition of pyruvate in samples collected 5-, 10-, 20- and 30-min reperfusion at 25 °C.

HK activity was measured in mitochondrial enriched samples at 25 °C with glucose-6-phosphate dehydrogenase, glucose, ATP and NAD^+^, in the presence of rotenone (1 μM) to inhibit NADH breakdown by oxidoreductase enzyme activity. Mitochondrial enriched samples were corrected for citrate synthase (CS), a mitochondrial marker. CS was determined at 25 °C using acetyl-CoA, oxaloacetate and di-thionitrobenzoic acid, measuring the formation of thionitrobenzoic acid at 412 nm.

### Cardiac energetics

Cardiac energetics was measured in Langendorff perfused rat hearts (*n* = 4–6 per group). Hearts were exposed for 20 min to 1 μM TAT-only or 200 nM TAT-HKII. Thereafter, for one group, hearts were snap frozen in liquid nitrogen, weighed and stored at −80 °C until analysis. Another group was exposed to 15-min ischemia followed by 7-min reperfusion, after which the hearts were frozen.

Hearts were freeze-dried overnight and dry weight was determined. ATP, PCr, creatinine (Cr) and inorganic phosphate (Pi) were determined as described by Fiolet et al. [9]. The phosphorylation potential (ΔG_ATP_) was calculated from these values.

### Statistical analysis

Data are presented as mean + SEM. Data were analysed by an independent *t* test when there were two groups, or by ANOVA followed by a Dunnet’s post hoc test with saline as control group when there were more than two groups. MVO_2_, RPP and MVO_2_/RPP at baseline were analysed by a two-way ANOVA for time and group, and Dunnet’s post hoc test when applicable. MitoPO_2_ was tested using a paired *t* test.

## Results

### 1 μM TAT-HKII causes ischemia

**Fig. 2:**
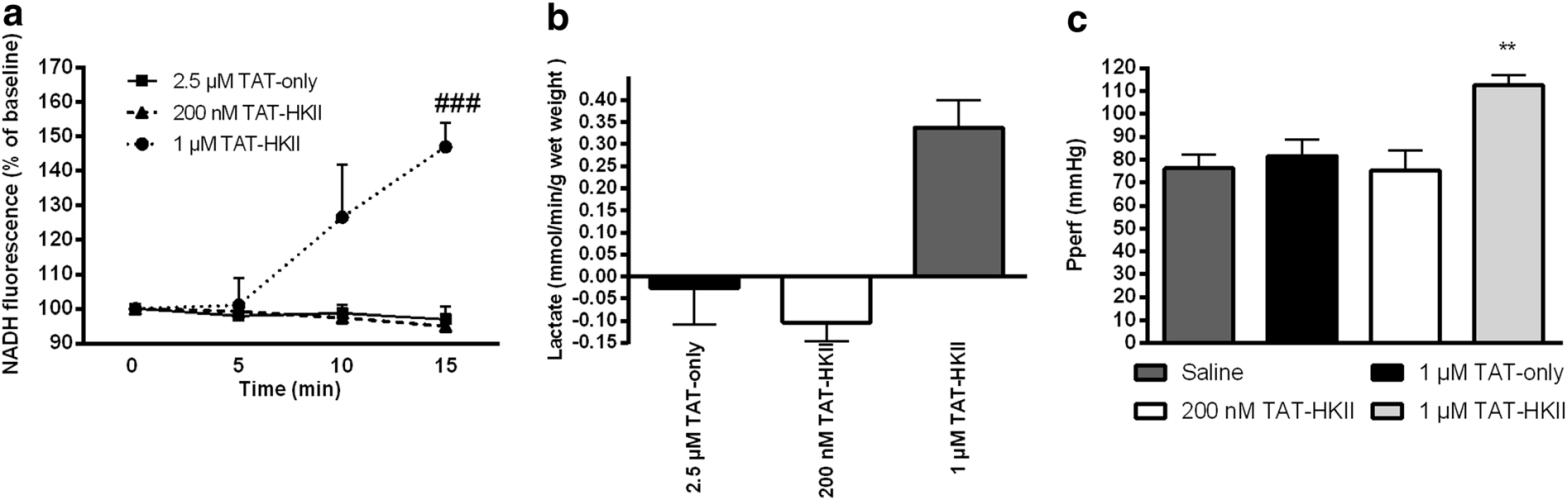
Low-dose TAT-HKII caused no ischemia, whereas higher-dose TAT-HKII caused modest ischemia. Hearts were treated for 15 min with saline, 2.5 μM TAT-only or 200 nM or 1 μM TAT-HKII. NADH fluorescence in the heart during the 15-min peptide treatment (**a**), lactate in effluent after 15-min peptide treatment (**b**) and perfusion pressure Pperf at the end of peptide treatment (**c**). ^###^ *p* < 0.001 vs 2.5 μM TAT-only; **p < 0.01 vs saline. Mean + SEM, *n* = 3–4 for **a** and **b**, *n* = 5–7 for **c**. Data for 2.5 μM TAT-only and 200 nM TAT-HKII in **a** and **b** have been published before [23] and are provided to facilitate direct comparisons with the 1 μM TAT-HKII peptide treatment

In TAT-only and 200 nM TAT-HKII-treated hearts, 15-min peptide treatment did not lead to an increase in NADH fluorescence (97 and 95% from baseline, respectively) (Fig. 2a; published previously [23]). In addition, both treatments did not lead to lactate production (Fig. 2b). However, 1 μM TAT-HKII treatment caused an increase in NADH fluorescence to 147% of baseline (Fig. 2a). This increase was significant when compared to TAT-only (*p* < 0.001). In addition, it non-significantly increased lactate production (*p* = 0.051) (Fig. 2b), and perfusion pressure was significantly increased to 112.6 mmHg after 20-min perfusion (*p* = 0.006) (Fig. 2c). These results indicate that 1 μM TAT-HKII treatment causes ischemia. Because this makes it difficult to distinguish the effects of HKII translocation and ischemia, we decided to only report the treatment groups (saline, TAT-only, 200 nM TAT-HKII) for which no signs of ischemia were detected.

### TAT-HKII decreases oxygen consumption but is without effect on cardiac economy before ischemia

**Fig. 3:**
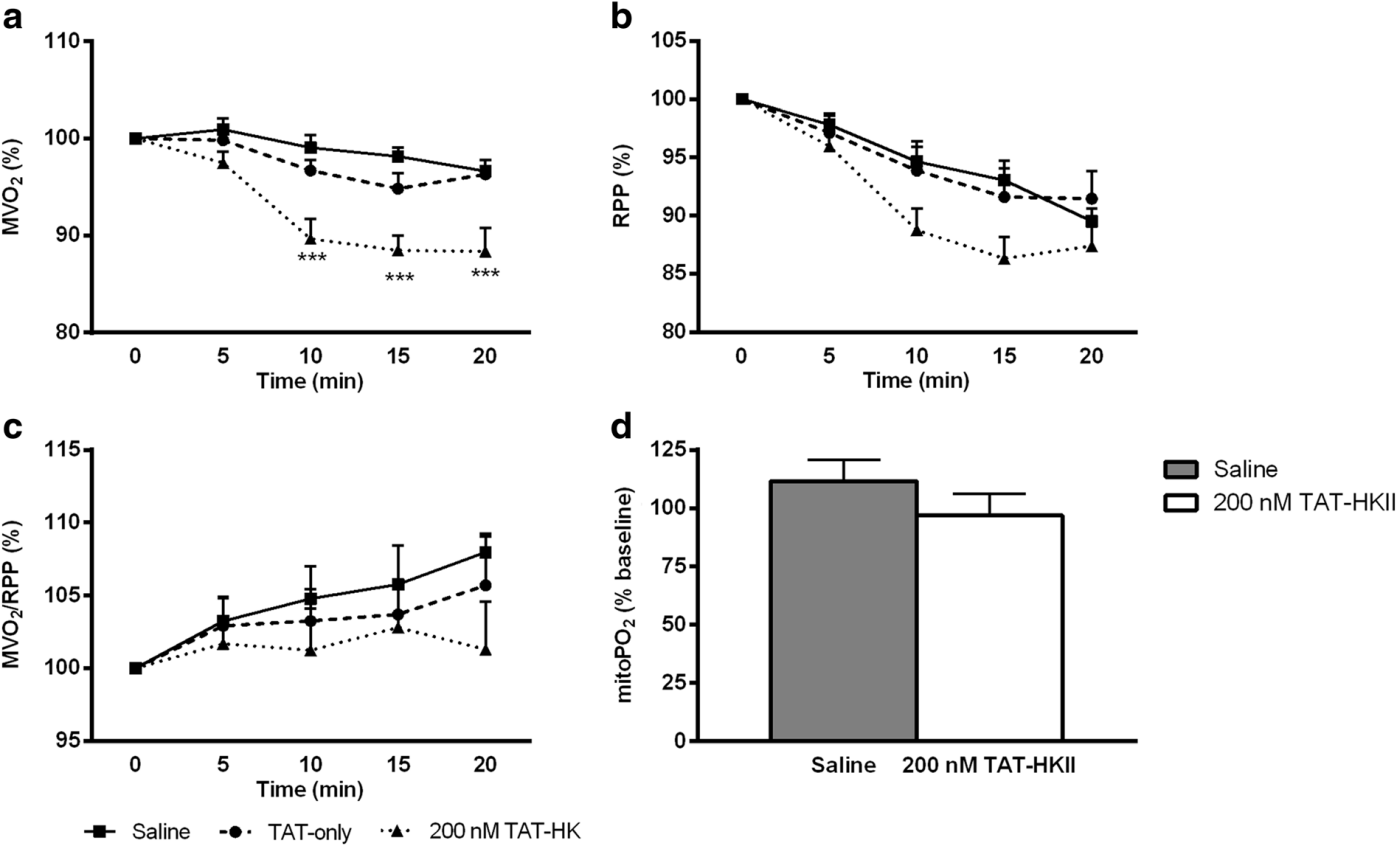
TAT-HKII treatment decreased cardiac oxygen consumption but was without effect on myocardial economy before ischemia. Langendorff perfused rat hearts were exposed to 20 min saline, 1 μM TAT-only or 200 nM TAT-HKII treatment. Cardiac oxygen consumption (MVO_2_) (**a**), cardiac function (rate pressure product (RPP)) (**b**) and myocardial economy (MVO_2_/RPP) (**c**) during Langendorff perfusion. Mitochondrial oxygen tension (mitoPO_2_) after 20-min peptide treatment as percentage of baseline (**d**). **p < 0.01 vs saline. Mean + SEM, *n* = 5–8 per group

In both control groups, MVO_2_ was decreased to 96% of baseline at the end of peptide treatment (Fig. 3a). In the 200 nM TAT-HKII group, this decrease was significantly larger (88% of baseline; *p* = 0.004). Cardiac function, as reflected by the rate-pressure-product, only showed a non-significant trend to decrease with TAT-HKII treatment (Fig. 3b). However, when corrected for RPP, MVO_2_ was not different in the 200 nM TAT-HKII group when compared to control groups (Fig. 3c), indicating that cardiac economy was unaltered.

Next to MVO_2_, mitoPO_2_ during peptide treatment was measured in a different set of experiments. Workload in these hearts was comparable to paced hearts (mean heart rate 277 bpm and RPP 35,384 bpm * mmHg). At baseline, there were no differences in mitoPO_2_ between groups (27.3 and 25.1 mmHg for saline and 200 nM TAT-HKII, respectively). TAT-HKII peptide treatment was not associated with a change in mean mitoPO_2_ (Fig. 3d), supporting our previous results (Fig. 2) that 200 nM TAT-HKII treatment was not associated with cardiac ischemia.

### A reduction in mtHKII turns reversible I/R injury in irreversible I/R injury

**Fig. 4:**
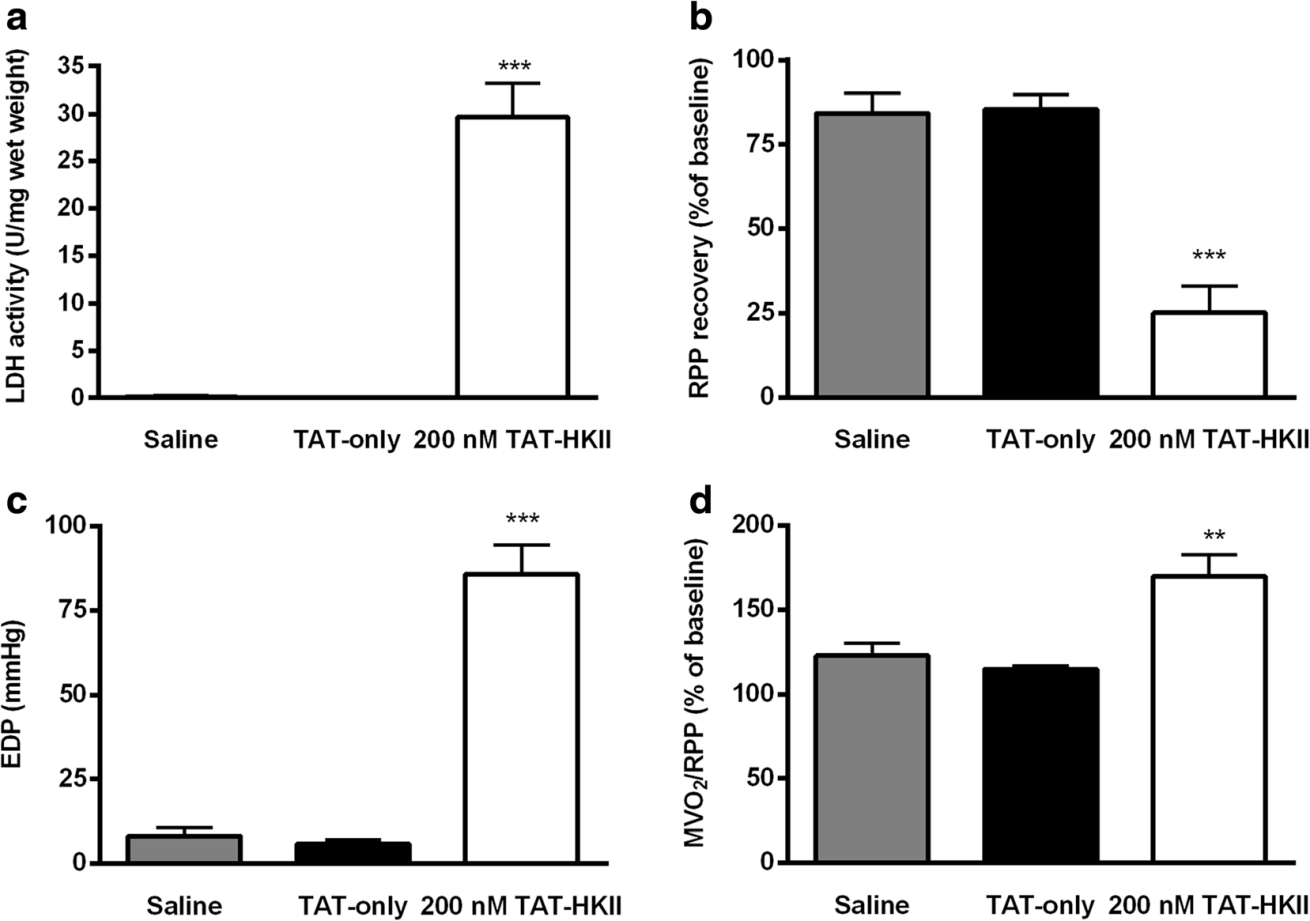
A reduction in mtHKII turned reversible I/R into irreversible I/R. Hearts were exposed to 20-min treatment with saline, 1 μM TAT-only or 200 nM TAT-HKII, followed by 15-min ischemia and 30-min reperfusion. Total lactate dehydrogenase (LDH) activity released during 30-min reperfusion (**a**), rate pressure product (RPP) (**b**), end-diastolic pressure (EDP) (**c**) and cardiac oxygen consumption (MVO_2_)/RPP (**d**) determined at 30-min reperfusion. **p < 0.01, ***p < 0.001 vs saline. Mean + SEM, *n* = 5–7 per group

At the end of reperfusion, both control groups showed no signs of I/R injury. Cell death was not observed (Fig. 4a), RPP returned to 84 and 85% of baseline (Fig. 4b) and end-diastolic pressure (EDP) returned to 8 and 6 mmHg (Fig. 4c) for the saline and 1 μM TAT-only group, respectively. When HKII was translocated from the mitochondria, extensive I/R injury was observed: hearts had a pronounced significant rise in LDH release (29.7 μmol/g heart weight/30-min reperfusion; *p* < 0.001), cardiac recovery was reduced to 25% and EDP rose to 86 mmHg (both *p* < 0.001). In addition, TAT-HKII treatment decreased cardiac economy at end reperfusion, as MVO_2_/RPP was significantly higher in TAT-HKII-exposed group (*p* = 0.001) (Fig. 4d), indicating uncoupling between oxygen consumption and force development.

### TAT-HKII increases ROS

**Fig. 5:**
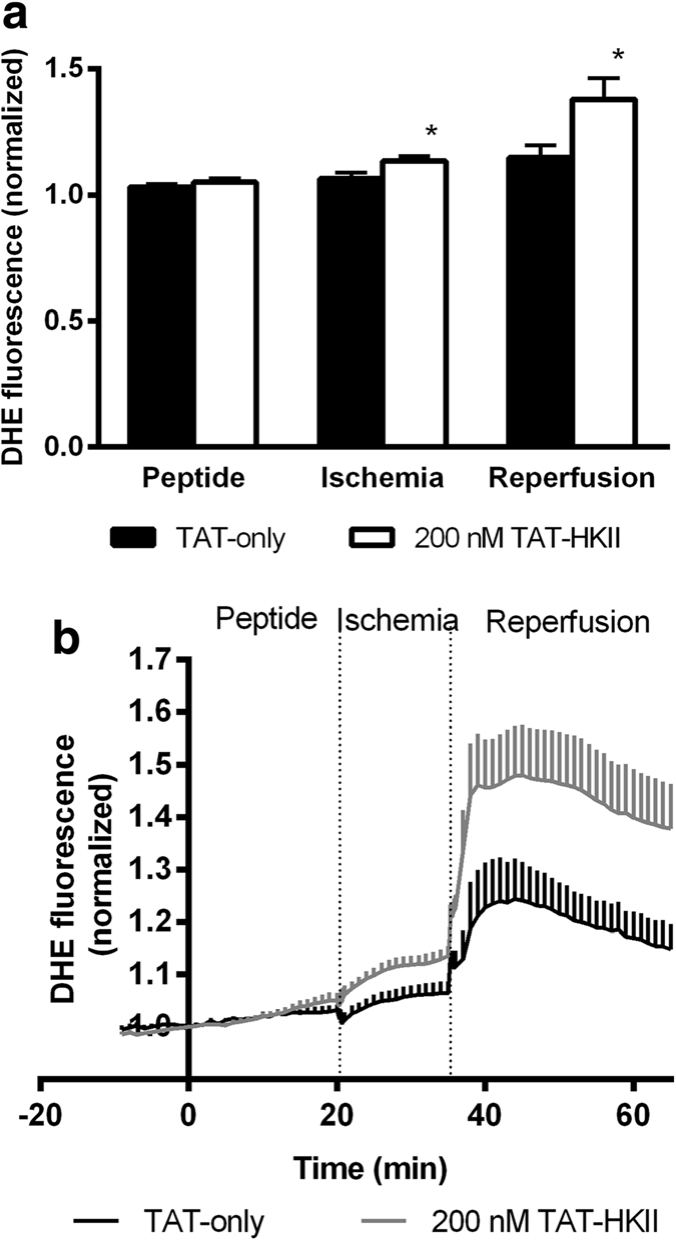
TAT-HKII treatment increased ROS production during ischemia and reperfusion. ROS levels before ischemia (peptide treatment) and during the I/R intervention (**a**), and ROS levels at the end of peptide treatment, at the end of ischemia and at the peak during early reperfusion (**b**). *p < 0.05 vs TAT-only. Mean + SEM, *n* = 6 per group

Shown in Fig. 5a are the ROS levels indexed by normalized DHE fluorescence in TAT-control and TAT-HKII-treated hearts before and during the I/R intervention. Also, quantified are the ROS levels at the end of baseline, the end of ischemia and the peak levels during early reperfusion (Fig. 5b). Before ischemia, there were no differences in ROS levels between groups. In contrast, TAT-HKII-treated hearts exhibited significantly greater increase in ROS levels at 15 min of ischemia (by 63%; *p* = 0.039) and at the peak during reperfusion (by 96%; *p* = 0.04) compared to control hearts. Of note, the difference in peak ROS levels between both groups was most pronounced during the reperfusion phase. No differences between the TAT control group and a saline group (*n* = 3) were observed (data not shown).

### TAT-HKII translocates HKII from mitochondria

**Fig. 6:**
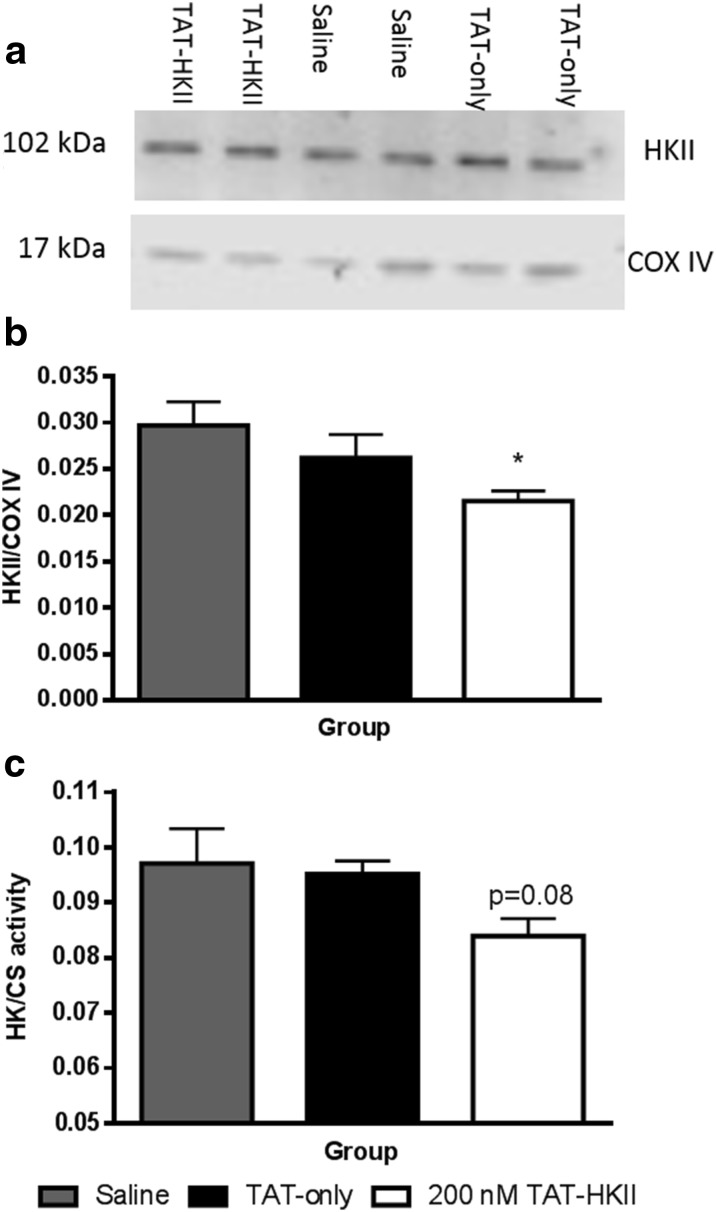
TAT-HKII treatment reduced mitochondrial bound hexokinase. Hearts were treated for 20 min with saline, 1 μM TAT-only or 200 nM TAT-HKII, exposed to 15-min ischemia and 7-min reperfusion. Representative images of the western blots (**a**), semi-quantitative amount of hexokinase II (HKII)/COX IV bound to the mitochondria as determined by western blot (**b**) and HK activity corrected for citrate synthase (CS) activity at 7-min reperfusion (**c**). *p < 0.05 vs saline. Mean + SEM, *n* = 6 per group

At 7 min of reperfusion, we determined the amount of HKII in our mitochondria-enriched fraction (Fig. 6a, b). TAT-HKII treatment significantly reduced mtHKII binding with 26% compared to saline (*p* = 0.03). Additionally, HK activity confirmed the results observed by western blot (Fig. 6c). HK/CS activity, indicating both HKI and HKII activity, was reduced 13% in animals treated with 200 nM TAT-HKII (*p* = 0.08). Knowing that HKI and HKII contribute approximately 50% to cardiac HK activity [34], the 13% of total HK activity then translates in a 26% reduction of HKII activity, commensurate with the 26% HKII protein reduction.

### TAT-HKII increased PCr before ischemia

**Fig. 7:**
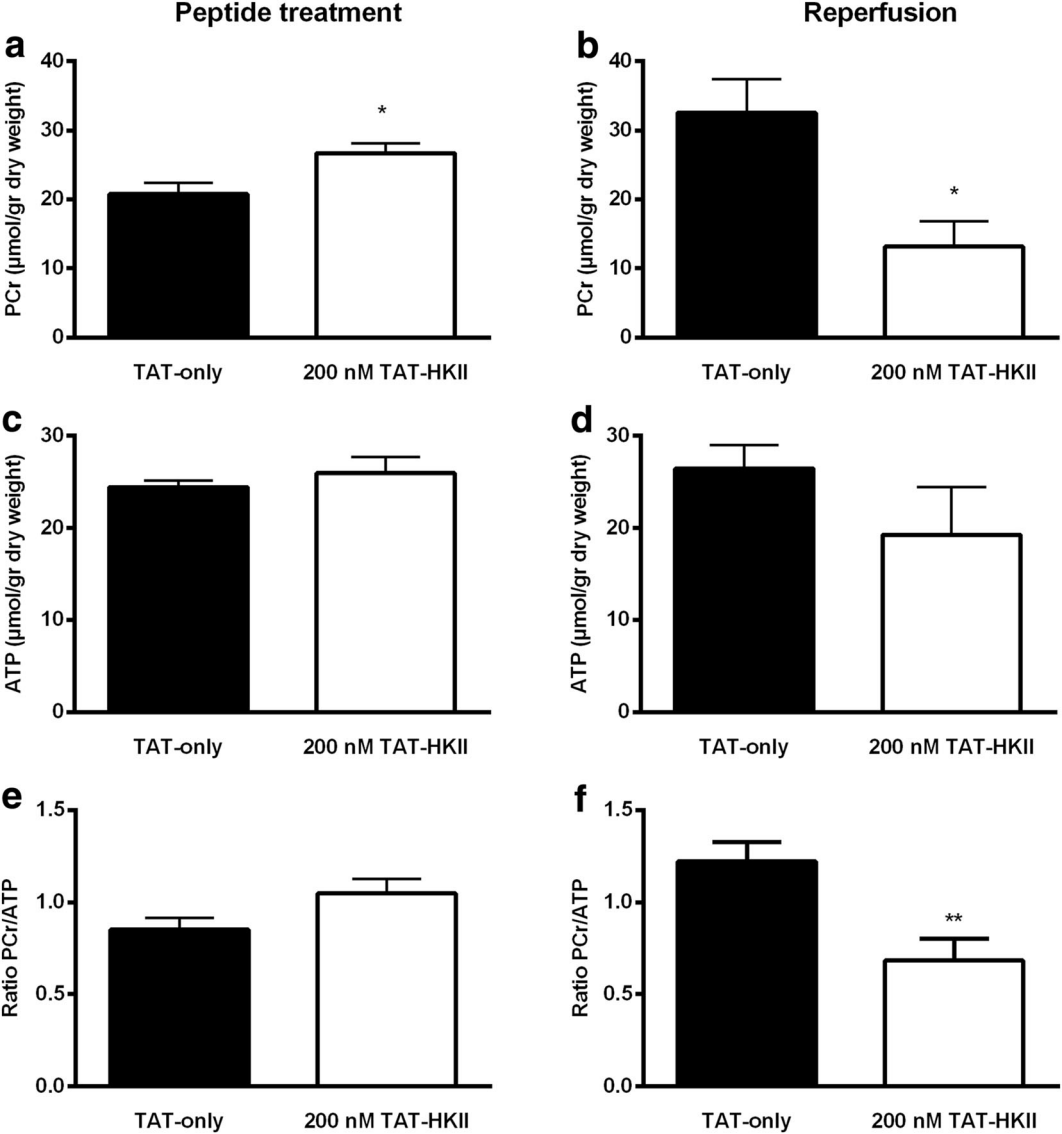
TAT-HKII increases PCr before ischemia, but lowers PCr after ischemia. Hearts were treated for 20 min with 1 μM TAT-only or 200 nM TAT-HKII. Energetics were measured at the end of peptide treatment (baseline) or after 15-min ischemia and 7-min reperfusion. Phosphocreatine (PCr) (**a**, **b**), ATP (**c**, **d**) and ratio PCr/ATP (**e**, **f**) at the end of 20-min treatment (**a**, **c**, **e**) and after ischemia (**b**, **d**, **f**). *p < 0.05, **p < 0.01 vs TAT-only at the same time-point. Mean + SEM, *n* = 4–6 per group

Twenty-minute treatment with 200 nM TAT-HKII significantly increased PCr when compared to TAT-only treatment (26.7 vs 21.5 μmol/g dry weight, respectively; *p* = 0.02) (Fig. 7a). In addition, 200 nM TAT-HKII non-significantly increased PCr/ATP ratio, a clinical indicator for the energy state of the heart, from 0.85 to 1.05 (*p* = 0.08) (Fig. 7e). No effects were observed on ATP (Fig. 7c), inorganic phosphate (Pi), creatine or free energy of ATP hydrolysis (ΔG_ATP_) (data not shown).

In contrast to the effects of peptide treatment before ischemia, after ischemia PCr and PCr/ATP were significantly lower with TAT-HKII treatment when compared to control (*p* = 0.02 and *p* = 0.01, respectively) (Fig. 7b, f). Also, after ischemia, no differences in ATP were observed (Fig. 7d).

## Discussion

The results of our study show that a loss of mtHKII can mediate the transition from non-injurious reversible I/R into one that is injurious, suggesting that the amount of HKII bound to mitochondria is a determinant of when I/R becomes damaging. This loss of mtHKII improved cardiac energetics and reduced oxygen consumption before I/R, negating the possibility that reduced mtHKII caused increased cardiac I/R injury due to impaired energetics or increased mitochondrial respiration. In contrast, decreasing mtHKII causes increased ROS production during the ischemia and reperfusion period, suggesting the involvement of mtHKII in I/R-induced ROS production and that increased ROS may be the trigger for injury to start occurring.

Previous studies have shown that a reduction in mtHKII can exacerbate I/R injury [28, 33, 39]. The amount of mtKII has been shown to correlate with infarct size [28], and a reduction in mtHKII has been shown to block IPC [33], decrease cardiac function and alter cardiac remodelling [39]. In addition, cardioprotective interventions, like insulin, morphine and IPC, are associated with an increase in mtHKII [41]. In this study, we observed that a reduction in mtHKII causes cell death, increases EDP and reduces RPP after 15-min ischemia. Factors are not affected in control hearts. The reduction observed in RPP in control hearts can be considered as normal deterioration over time in a Langendorff system [42]. This is the first study showing that a reduction in mtHKII can also turn a non-injurious episode of 15-min ischemia into an injurious ischemia.

This switch from non-injurious to injurious ischemia might have been caused by an increase in ROS production during ischemia and reperfusion after HKII translocation. Multiple studies have shown that a reduction in mtHKII increases ROS production [7, 32, 40], while an increase in mtHKII can reduce ROS [25, 37]. ROS is an important factor in opening of the mitochondrial permeability transition pore (MPTP) [13]. Dimerization of F_0_F_1_ATPase in the mitochondrial membrane has recently been proposed as molecular identity of the MPTP [11]. The MPTP opens when matrix Ca^2+^ and ROS levels are high [13]. When the MPTP opens, molecules <1.5 kDa can passage the mitochondrial membrane, finally leading to cell death. The increase in ROS production after HKII translocation might have caused increased MPTP opening and thereby increased cell death. This study provides evidence that also after a short period of ischemia, a reduction in mtHKII causes an increase in ROS and might thereby increase cardiac cell death.

ROS may be increased during ischemia and reperfusion after mtHKII detachment because cytochrome c (cyt c) is released from the intermembrane space. Previously, we showed that cytosolic cyt c is significantly higher in reperfused hearts after treatment with 200 nM TAT-HKII [33]. Cyt c is a powerful superoxide scavenger present in the inner mitochondrial membrane. MtHKII forms mitochondrial contact sites with other proteins. Disruption of HKII from these contact sites might increase permeability of the OMM, as it does in reconstituted vesicles [1]. A translocation of HKII from the mitochondria, in combination with increased Ca^2+^ levels, disrupts contact sites and increases permeability of the OMM causing cyt c to be released [27, 33]. This will result in increased ROS.

Recently, it has become clear that respiratory inhibition is a nodal point for several cardioprotective interventions (nitrosylation, IPC, hypothermia, respiratory inhibitors) through which protection against I/R injury is conferred. Previous research also indirectly indicated that increasing mtHK would slow down mitochondrial metabolism [12, 22, 42, 43]. We therefore anticipated that reduced mtHKII would result in increased oxygen consumption and thereby contribute to increased I/R injury. However, our results during baseline clearly show this not to be the case: TAT-HKII actually decreased MVO_2_ in the glucose-only perfused rat heart. However, since cardiac function also decreased, no changes in cardiac economy were observed. Whether similar effects can be observed in hearts perfused with different substrates will have to await further studies. In addition, our results negate the possibility that the transition to injurious I/R injury with TAT-HKII peptide treatment is mediated through increased mitochondrial respiration. The decreased MVO_2_ during baseline and the increased economy after I/R might indicate mitochondrial uncoupling.

The results also exclude a role for impaired energetics as mechanism provoking injurious I/R injury upon loss of mtHKII. The increased PCr at baseline is likely a consequence (secondary effect) of the reduction in cardiac oxygen consumption: the heart’s energy turnover is decreased, resulting in a more energetic state of the heart [8, 38]. In addition, the lower PCr levels after ischemia are probably not caused by a direct effect of the reduction in mtHKII, but indirectly by cell death in these hearts.

During reperfusion, PCr in control hearts is higher than at baseline. It is known that during reversible ischemia, PCr levels decrease and quickly recover during reperfusion to levels higher than before ischemia. This phenomenon is known as ‘PCr overshoot’ [14]. The presence of PCr overshoot is another sign that the 15-min ischemia did not lead to irreversible damage in control hearts [36].

## Methodological considerations

In this study, we planned to use two different concentrations of the TAT-HKII peptide. A low dose of 200 nM that was previously shown by us to decrease mtHKII without causing ischemia [23, 33]. The higher dose of 1 μM was chosen to study larger effects of a loss of mtHKII. However, after start of the experiments, Pasdois et al. suggested that the TAT-HKII peptide impairs vascular function and that some of its effects may be mediated by vasoconstriction of the coronary vasculature and developing ischemia rather than dissociation of HKII from the mitochondria [26]. We showed that the low dose of 200 nM TAT-HKII did not cause any ischemia and that effects observed by higher doses of the TAT-HKII peptide cannot be explained by ischemia alone [23]. We did, however, observe that 2.5 μM TAT-HKII increased NADH epifluorescence and increased lactate production, indicating that it causes minor ischemic damage. To exclude a role of ischemia in this study, we also measured lactate and NADH epifluorescence after administering 1 μM TAT-HKII, and we observed an increase in both parameters. Because it would be difficult to separate the effects caused by the HKII translocation and the ischemia caused by the peptide, we decided to continue with the 200 nM TAT-HKII dose only.

The protocol used to isolate mitochondria does not lead to isolation of a pure mitochondrial fraction, but a mitochondrial enriched fraction. A more elaborate isolation protocol would have led to HK dissociation from the mitochondria [16, 31]. HK in the heart is either located at the mitochondria or in the cytosol [4, 35], and therefore, the use of a more elaborate protocol was not necessary.

Finally, this study only looked at necrotic cell death and not apoptosis, since its role is only significant after a longer period of ischemia and/or reperfusion [10, 17]. In acute I/R injury, necrosis has been observed as the main contributor to myocardial cell death [15, 17].

## Conclusions

The present study shows that the loss of mtHKII can be a master switch turning reversible ischemia into irreversible ischemia. This loss of mtHKII is associated with increased ROS production after ischemia, but not with increased respiratory activity or decreased cardiac energetics at baseline.
